# Rehearsal simulation to determine the size of device for left atrial appendage occlusion using patient-specific 3D-printed phantoms

**DOI:** 10.1038/s41598-022-11967-2

**Published:** 2022-05-11

**Authors:** Dayeong Hong, Sojin Moon, Youngjin Cho, Il-Young Oh, Eun Ju Chun, Namkug Kim

**Affiliations:** 1 Department of Biomedical Engineering, Asan Medical Institute of Convergence Science and Technology, Asan Medical Center, University of Ulsan College of Medicine, Seoul, 05505 South Korea; 2 Division of Cardiology, Department of Internal Medicine, Seoul National University, Bundang Hospital 82, Gumi-ro 173beon-gil, Bundang-gu, Gyeonggi-do, Seongnam-si, 13620 South Korea; 3 Department of Radiology, Seoul National University Bundang Hospital 82, Gumi-ro 173beon-gil, Bundang-gu, Gyeonggi-do, Seongnam-si, 13620 South Korea; 4 Department of Radiology and Convergence Medicine, University of Ulsan College of Medicine, Asan Medical Center, 388-1 Pungnap2-dong, 88 Olympic-ro 43 Gil, Songpa-gu, Seoul, 05505 South Korea

**Keywords:** Interventional cardiology, Cardiovascular diseases

## Abstract

Left atrial appendage (LAA) occlusion (LAAO) is used to close the finger-like extension from the left atrium with occlusion devices to block the source of thrombosis. However, selection of the devices size is not easy due to various anatomical changes. The purpose of this study is patient-specific, computed tomography angiography (CTA)-based, three-dimensionally (3D) printed LAAO phantoms were applied pre-procedure to determine the size. Ten patients were enrolled prospectively in March 2019 and December 2020. The cardiac structure appearing in CTA was first segmented, and the left atrium and related structures in the LAAO procedure were modeled. The phantoms were fabricated using two methods of fused deposition modeling (FDM) and stereolithography (SLA) 3D printers with thermoplastic polyurethane (TPU) and flexible resin materials and evaluated by comparing their physical and material properties. The 3D-printed phantoms were directly used to confirm the shape of LAA, and to predict the device size for LAAO. In summary, the shore A hardness of TPU of FDM was about 80–85 shore A, and that of flexible resin of SLA was about 50–70 shore A. The measurement error between the STL model and 3D printing phantoms were 0.45 ± 0.37 mm (Bland–Altman, limits of agreement from − 1.8 to 1.6 mm). At the rehearsal, the estimations of device sizes were the exact same with those in the actual procedures of all 10 patients. In conclusion, simulation with a 3D-printed left atrium phantom could be used to predict the LAAO insertion device size accurately before the procedure.

## Introduction

The left atrial appendage (LAA) occlusion (LAAO) closes the entrance of the left atrial appendage using occlusion devices to block the source of thrombosis as 90% of the thrombus is generated in the left atrium^1–3^. The treatment of patients with atrial fibrillation has relied on anticoagulants to prevent thrombosis and stroke^4–6^. However, patients with old age or chronic illnesses have to take lifelong medication and experience side effects, such as bleeding^7,8^. Therefore, LAAO has been proposed as a new treatment for atrial fibrillation patients. LAAO is a new therapeutic alternative to long-term anticoagulation^9–12^. The existing LAAO method was shown to be able to confirm the size of the LAA and predict the device in 3D LAA mapping and transesophageal echocardiography (TEE) of computed tomography (CT) angiography (CTA)^13,14^. In addition, due to the various morphologies, including cactus, chicken-wing, windsock, and cauliflower, current solutions are not sufficient, and leakage often occurs after the procedure^15–19^.

3D printing has been used to make a variety of medical models from medical images for procedural planning, education, simulation, and training^20–36^. Recently, the patient-specific model using 3D printing has actively been conducted. Of these, patient-specific 3D printing-based surgical repair of complex thoracoabdominal aortic disease as reported by Kim et al. may be a useful approach to enhance procedural efficiency^30^. Hong et al. developed a personalized and realistic educational thyroid cancer phantom based on CT images that supplements the training of residents learning to perform thyroid surgery^28^. Furthermore, Ko et al. developed a patient-specific breast surgery guide model for surgery planning and resident and patient education^37^. In addition, there have been 3D printing studies related to LAAO^38–40^. However, due to the limitations of 3D printing materials, it was difficult to implement a phantom with properties similar to the handling of a cardiologist during an actual procedure.

The purpose of this study was to propose patient-specific 3D printing technology suitable for LAAO with CTA and to confirm the medical education value of the proposed method.

## Methods

This study was conducted in accordance with the principles of the Declaration of Helsinki and current scientific guidelines. The study protocol was approved by the Seoul National University Bundang Hospital Institutional Review Board (IRB-B-2106/688-103). All of the methods were performed in accordance with the relevant guidelines and regulations.

**Figure 1:**
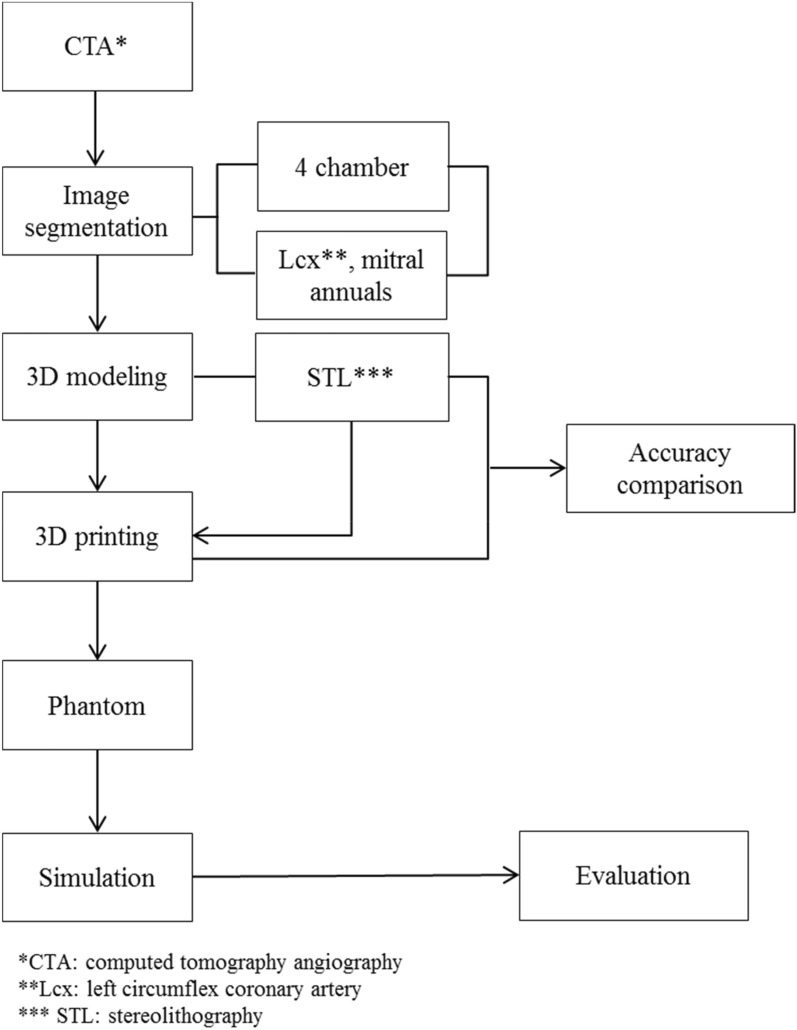
Overall workflow for developing rehearsal phantom for LAAO with 3D printing and medical images. *3D* three-dimensional, *CTA* computed tomography angiography, *LAAO* left atrial appendage occlusion, *Lcx* left circumflex coronary artery, *STL* stereolithography.

Based on medical images, such as CT and magnetic resonance imaging to make patient-specific 3D-printed phantoms, anatomical structures should be segmented and modeled to produce patient-specific 3D-printed phantom. Two types of 3D printers were used to fabricate actual phantoms with different materials. Shape accuracies and mechanical properties were evaluated to determine the final phantom material, which was evaluated through simulation. The stereolithography (SLA) 3D printer was used to fabricate actual phantoms with resin materials. It was applied to a rehearsal simulation of a total of 10 patients using phantom. The overall procedure is shown in Fig. 1.

### 3D-printing workflow

The procedure for fabricating 3D-printed rehearsal phantoms consists of multiple steps: (a) acquisition of a high-quality medical image of the anatomical structure, (b) medical image processing to extract the related regions of interest, (c) 3D modeling to accommodate the unmet clinical needs, (d) quality check and determination of the accuracy of the 3D printed phantom, (e) selection of 3D printing type and materials, and (f) printing the phantom.

In order to make good use of the diversity of 3D printing technology, designing and planning to accommodate the unmet clinical needs is important. The material is different depending on the type of 3D printing technology, which lead to the different mechanical properties of the phantom. A summary of the features and material properties of both printing techniques is shown in Table 1.

**Table 1:** Descriptions of two types of 3D printing techniques, including FDM and SLA.

Printing type	Additive manufacturing process
FDM	FDM technology constructs objects layer-by-layer from the bottom up by heating and extruding thermoplastic filament. The process is somewhat similar to SLA, and specialized programs, or slicers, “cut” CAD models into layers and compute the manner in which the printer's extruder should assemble each layer
SLA	SLA is a form of 3D printing technology used for producing models, prototypes, patterns, and creating parts layer by layer using photochemical processes by which light causes chemical monomers and oligomers to cross-link together to create polymers. This 3D printing type is quick and can make multi-designs; as such, it can be more expensive than FDM

*3D* three-dimensional, *CAD* computer-aided design, *FDM* fused deposition modeling, *SLA* stereolithography.

### CT acquisition

The cardiac CTAs of patients with various diseases requiring LAAO were scanned with dual-layer spectral-detector CT (IQon Spectral CT ®, Philips Healthcare, Best, The Netherlands) according to the standard protocol of Seoul National University Bundang Hospital (Seongnam, Republic of Korea). The CT scans were acquired at 120 kVp with 0.67-mm slice thickness. In addition, images were reconstructed to 0.3-mm axial sections using image reconstruction software (Spectral 3, Filter B, Philips, Best, The Netherlands). The data included the entire cardiac structure with the accompanying vessels.

### Anatomical design

**Figure 2:**
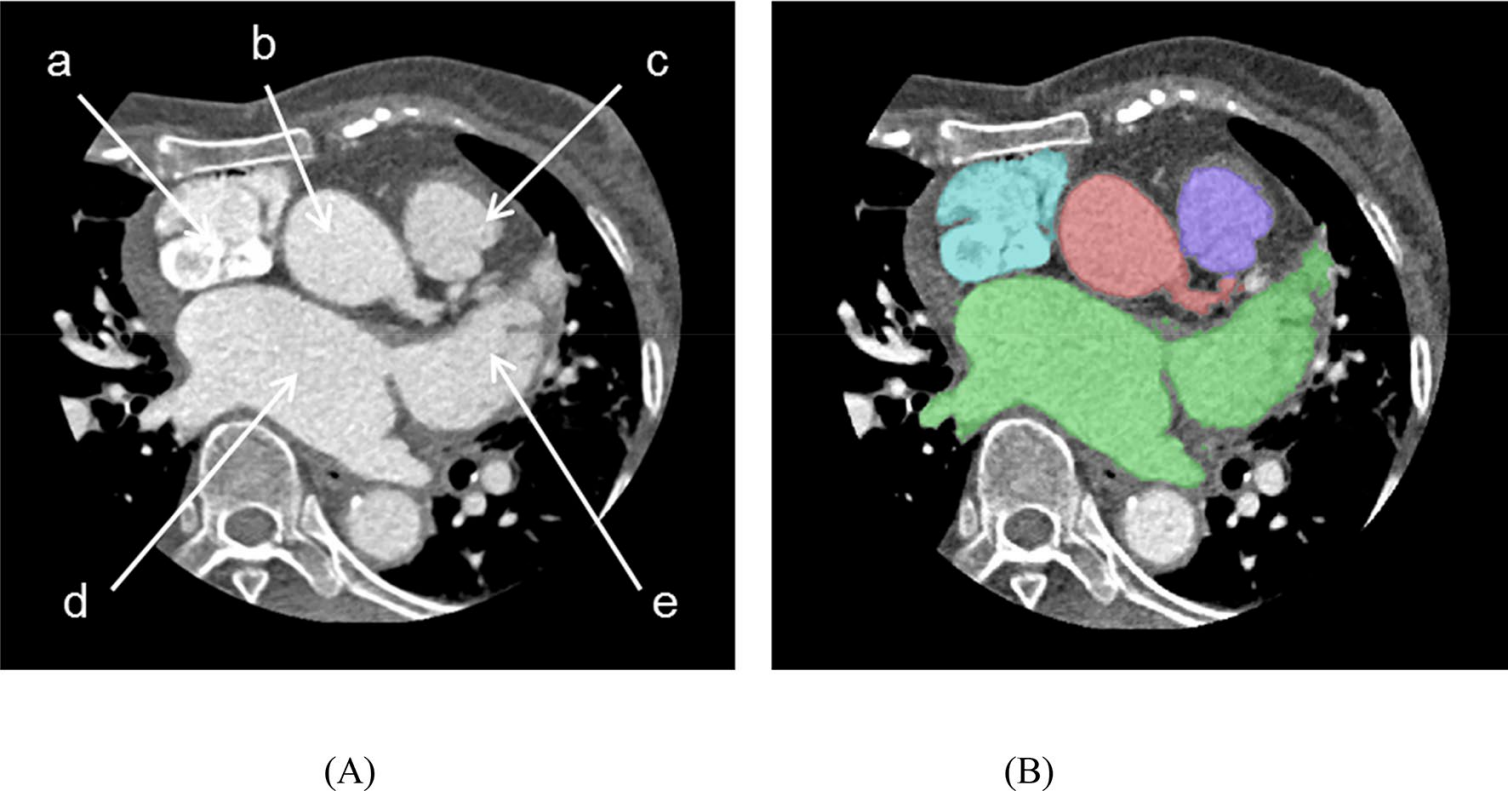
Segmentation based on cardiac anatomy by cardiac CT angiography, including: (**A**) CT image based cardiac anatomy: (**a**) cavoatrial junction, (**b**) ascending aorta, (**c**) main pulmonary artery, (**d**) left atrium, (**e**) left atrial appendage; and (**B**) Segmentation based on CT angiography. *CT* computed tomography (Spectral 3, Filter B, Philips, Best, The Netherlands).

Modeling specific structures related LAAO is very important regarding the procedure, which is a vascular intervention, and a catheter is inserted into the patient's femoral vein to reach the left atrium. In particular, it is not open surgery; thus, it is important to determine the location of the LAA and its surrounding structures. The important structures for the LAAO rehearsal phantoms include the right atrium, left atrium, aorta, left superior pulmonary vein, LAA, left circumflex coronary artery, and mitral annulus (Fig. 2). The four cardiac chambers, which have a relatively clear morphology, were easily segmented using the cardiac CT function of Mimics software. In contrast, the mitral annulus and left circumflex coronary artery, which do not exhibit clear shapes on the CT, were modeled by referring to the anatomical location.

### 3D printing with different materials

**Figure 3:**
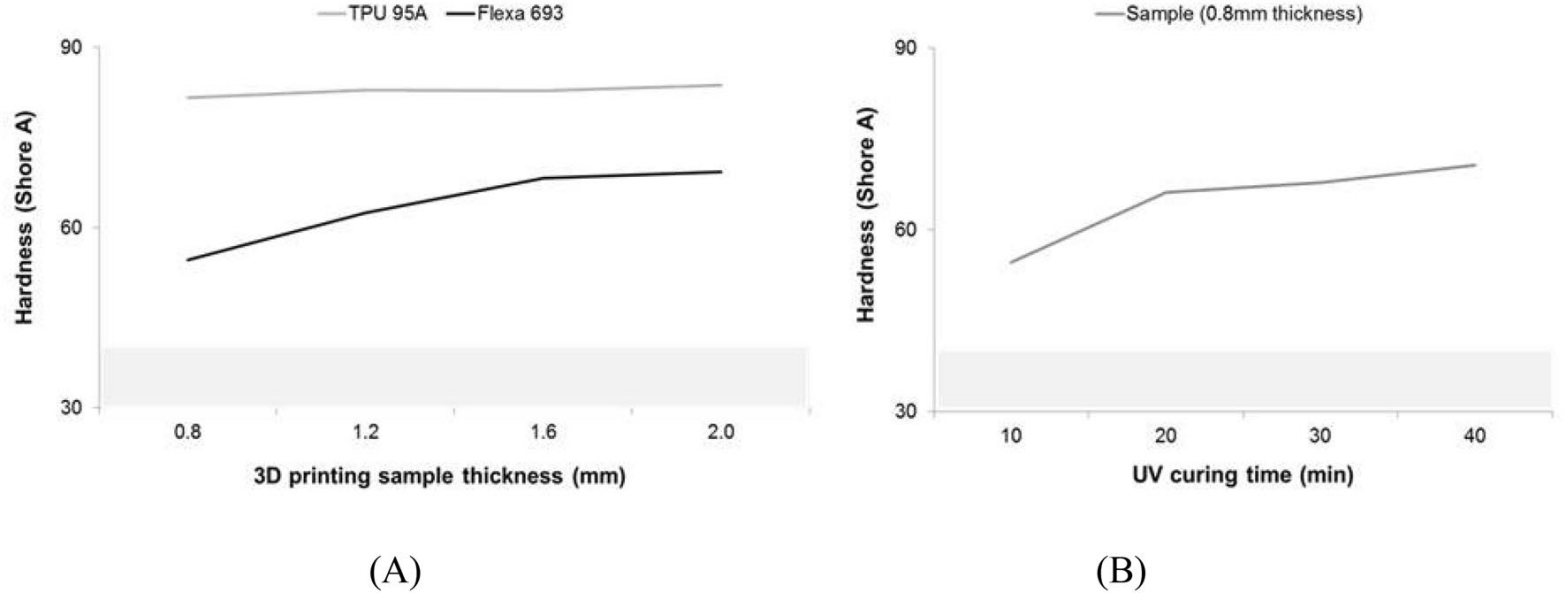
Comparison of mechanical properties of 3D printing materials. The gray zone in both graphs is human cardiac tissue to shore A hardness (mean) of about 40. (**A**) Hardness with different thickness values of two FDM printing materials. (**B**) Hardness according to UV curing time of one material. *3D* three-dimensional, *FDM* fused deposition modeling, *UV* ultraviolet.

The pilot study to determine 3D printing technology and materials was conducted to produce the rehearsal simulation phantoms. Thermoplastic polyurethane (TPU) material of fused deposition modeling (FDM) printer and flexible resin of SLA printer were printed with thicknesses of 0.8, 1.2, 1.6, and 2.0 mm. The size of the specimen was manufactured to be 3.0 × 3.0 mm, and, using a hardness tester, one researcher measured thrice for four locations to obtain an average value. As a result of measuring 95A shore hardness by thickness using the TPU material of the FDM printer, all of the specimens with a thickness of 0.8–2.0 mm were in the range of about 80–85 shore A. While measuring the hardness by thickness using the photopolymer resin of the SLA printer, the specimen with a thickness of 0.8–2.0 mm was in the range of 50–70 shore A. The result of the hardness measurement according to the ultraviolet (UV) curing time of the photopolymer resin was 54.6 shore A in 10 min. The hardness increased as UV was provided for more time. Although both materials were not within the range of actual human heart properties (which are less than 40), the 0.8-mm specimen, which is the smallest printable thickness of the SLA type, was the closest to the range of human heart properties (Fig. 3).

**Figure 4:**
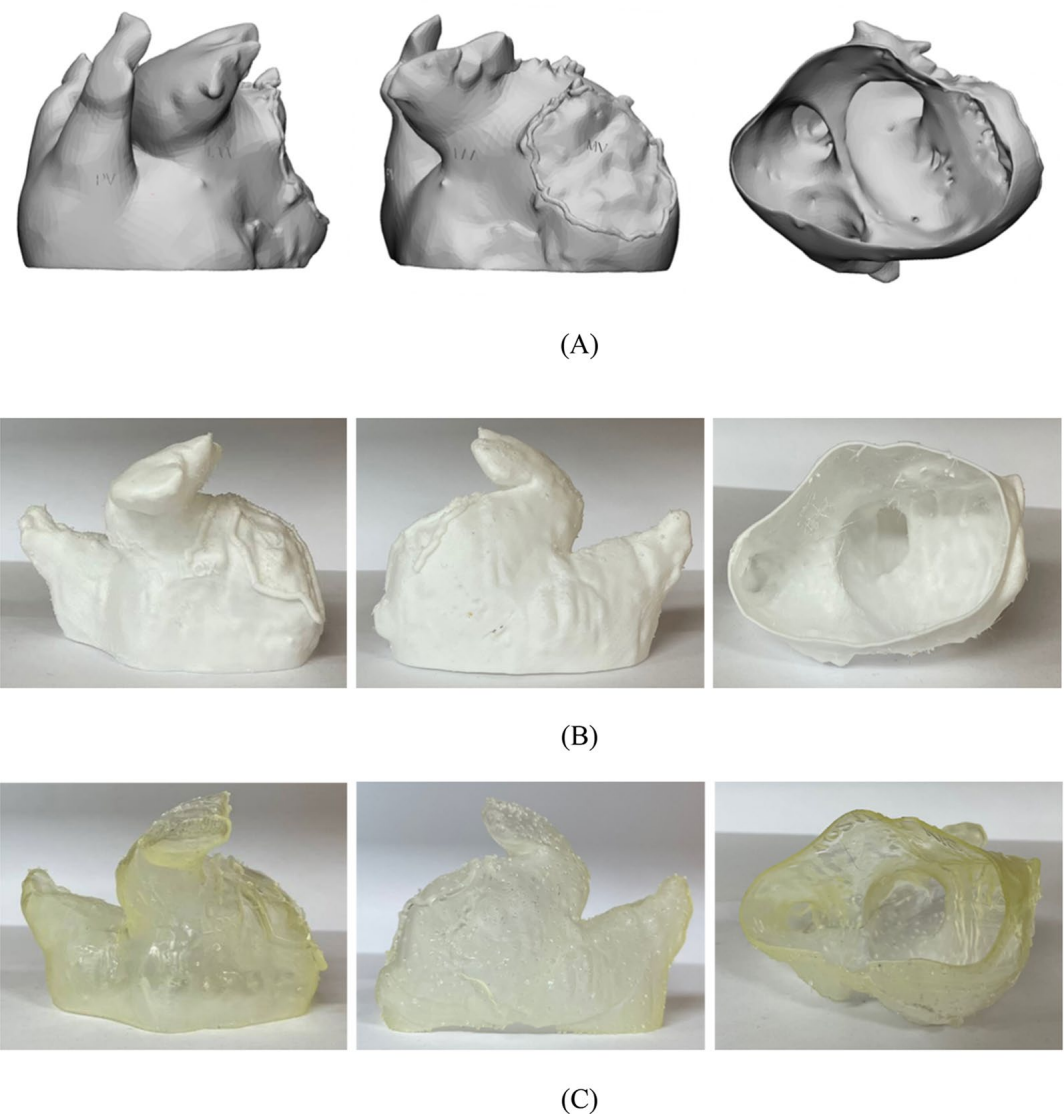
LAAO phantom made with two types of 3D printers. (**A**) 3D modeling (**B**) FDM, (**C**) SLA. *3D* three-dimensional, *FDM* fused deposition modeling, *LAAO* left atrial appendage occlusion, *SLA* stereolithography.

The LAAO phantom was printed using both materials for the material test. One out of 10 patients enrolled in the study was randomly selected. For the patient, phantoms were printed using FDM and SLA 3D printers (Fig. 4). Using the 3D-printed models with different materials, the LAAO rehearsal simulation phantoms were produced. The two printers are often used for medical printing due to their inexpensiveness and easy accessibility (Table 2). The FDM printer is highly commercialized and uses various types of materials, which are inexpensive compared with those used by other types of 3D printers. However, the hardness of these materials is difficult to control, and the surface is not smooth because the supporter is needed to overcome the inertia. On the other hand, SLA printers require more expensive materials compared with FDM printers, but the accuracy and surface smoothness are much higher. In addition, the hardness and transparency of the printout can be adjusted according to the printout thickness and UV curing conditions of the SLA printer.

**Table 2:** Comparison of two types of 3D printing, including FDM and SLA.

	FDM	SLA
Cost	Low cost	Relatively high cost
Materials	Thermoplastic	Photopolymer
Feature	Opacity, multi-color, need to support	Transparency, soft, high accuracy

*3D* three-dimensional, *FDM* fused deposition modeling, *SLA* stereolithography.

As an FDM printer, the Ultimaker S5 (Ultimaker BV, Geldermalsen, The Netherlands) was used with TPU 95A filament. Because the TPU 95A filament used in simulator production has a higher flexibility and elasticity compared with existing acrylonitrile butadiene styrene (ABS) and polylactic acid (PLA), TPU 95A was chosen among the various FDM filaments.

As an SLA printer, the X-Fab (DWS, Vicenza, Italy) was used with Flexa693, a photopolymer resin that has more flexibility and transparency. In addition, modifying the UV curing time of this SLA printer could control the elongation and hardness of the printout. Moreover, unlike FDM, the transparent material could be used for translucent printout.

### Procedure for printing 3D rehearsal phantom

Figure 1 shows the overall procedure for printing the LAAO rehearsal phantoms. Materials for phantom production were selected through material property tests. The final phantoms of 10 patients were produced by the SLA printer with Flexa693.

The cardiac CTA images were segmented and modeled using the medical image processing softwares Mimics and 3-matics (Materialise, Leuven, Belgium). The segmentation results were confirmed by a radiologist and a cardiologist independently, each with more than 15 y of experience. This segmentation took less than 1 h by an operator (D.H.), not counting the time it took to update the segmentation as requested by a cardiologist.

Major anatomical structures related to LAAO were segmented and modeled for the phantom making. Each anatomical structure was segmented directly from patient-specific CT data. The modeled 3D images were converted into stereolithography (SLA) format, consisting of a triangular surface mesh structure, by the software. In addition, it was printed using the XFAB, the SLA 3D printer. Printing time varied from person to person, but most LAAO models took about 5 h to print. Post-processing took 1–2 h. This was because in the case of the SLA type, an isopropanol (purity grade > 99.9%) cleaning process and an UV curing process were added to wash the resin after printing. Isopropanol washing took 15 min and UV curing took 10 min at 60°.

### Accuracy comparison between the STL model and the 3D-printed phantoms

**Figure 5:**
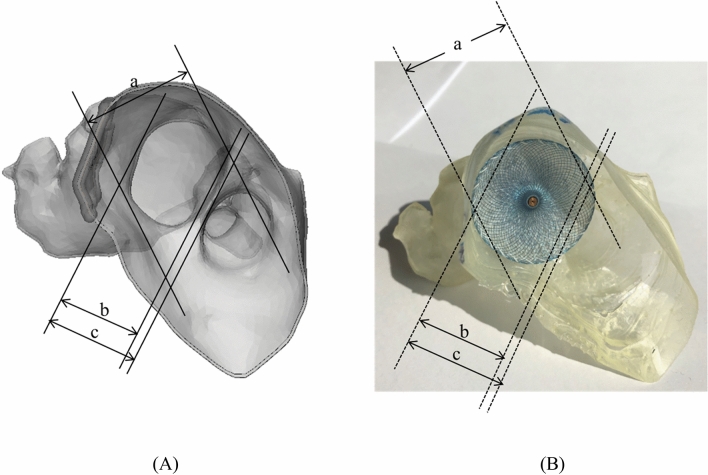
Measurements of shape accuracy between 3D model and 3D-printed phantom. (**A**) The 3D model (STL) with three landmarks specified for evaluating measurement error. (**B**) The 3D-printed phantom with three landmarks specified for evaluating measurement error. (**a**, diameter of the horizontal zone; **b**, LAA ostium; **c**, vertical zone). *3D* three-dimensional, *LAA* left atrial appendage, *STL* stereolithography.

**Figure 6:**
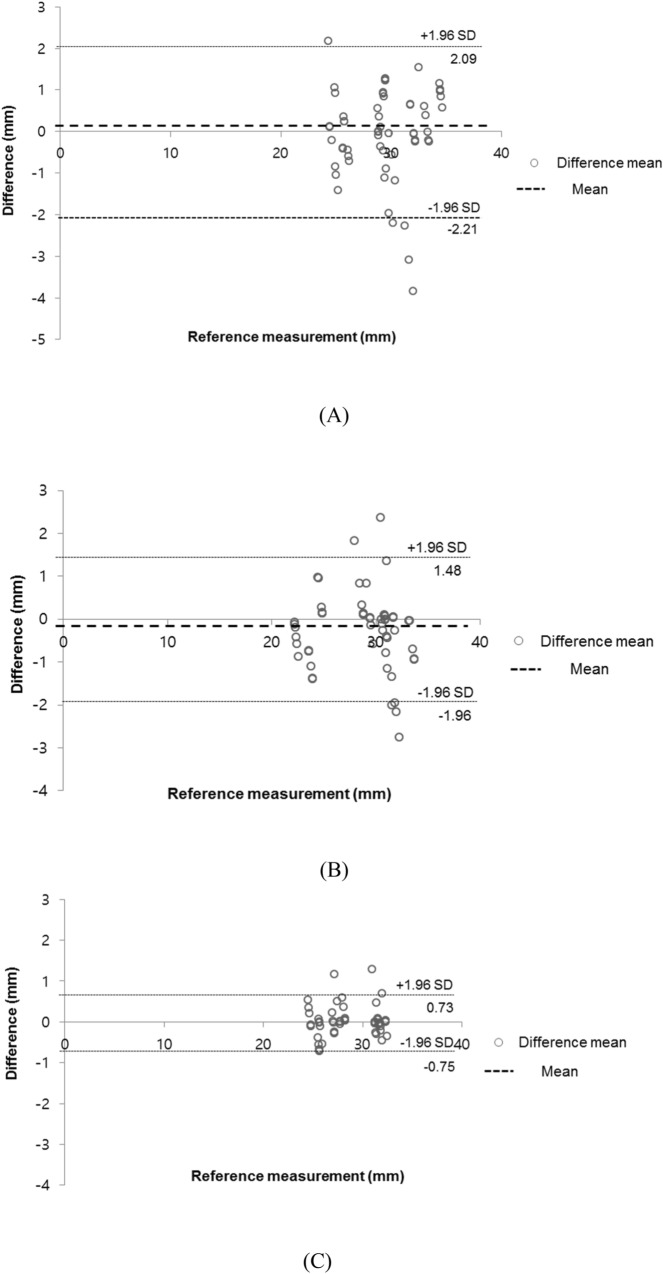
The Bland–Altman analysis to evaluate differences between the 3D model (STL) and the 3D-printed phantom. (**A**) Diameter of the horizontal zone, (**B**) LAA ostium, (**C**) Vertical zone. (SPSS version 25.00; IBM Corp., Armonk, NY, USA). *3D* three-dimensional, *STL* Stereolithography, *LAA* left atrial appendage.

The 3D-printed phantom was printed based on the STL model. To compare the accuracy of each 3D-printed phantom with that of the STL model, the same landmarks of three different locations were measured by two observers. In addition, each observer measured each of them thrice, for a total of 180 times (Fig. 5), using Vernier calipers. A Bland–Altman analysis was used to evaluate the accuracies between the STL model and printed phantom (Fig. 6). Paired t-tests were performed to statistically compare the differences between the STL model and the 3D-printed phantom using the SPSS software (version 25.00; IBM Corp., Armonk, NY, USA).

### Rehearsal simulation with 3D printed LAAO phantom

**Figure 7:**
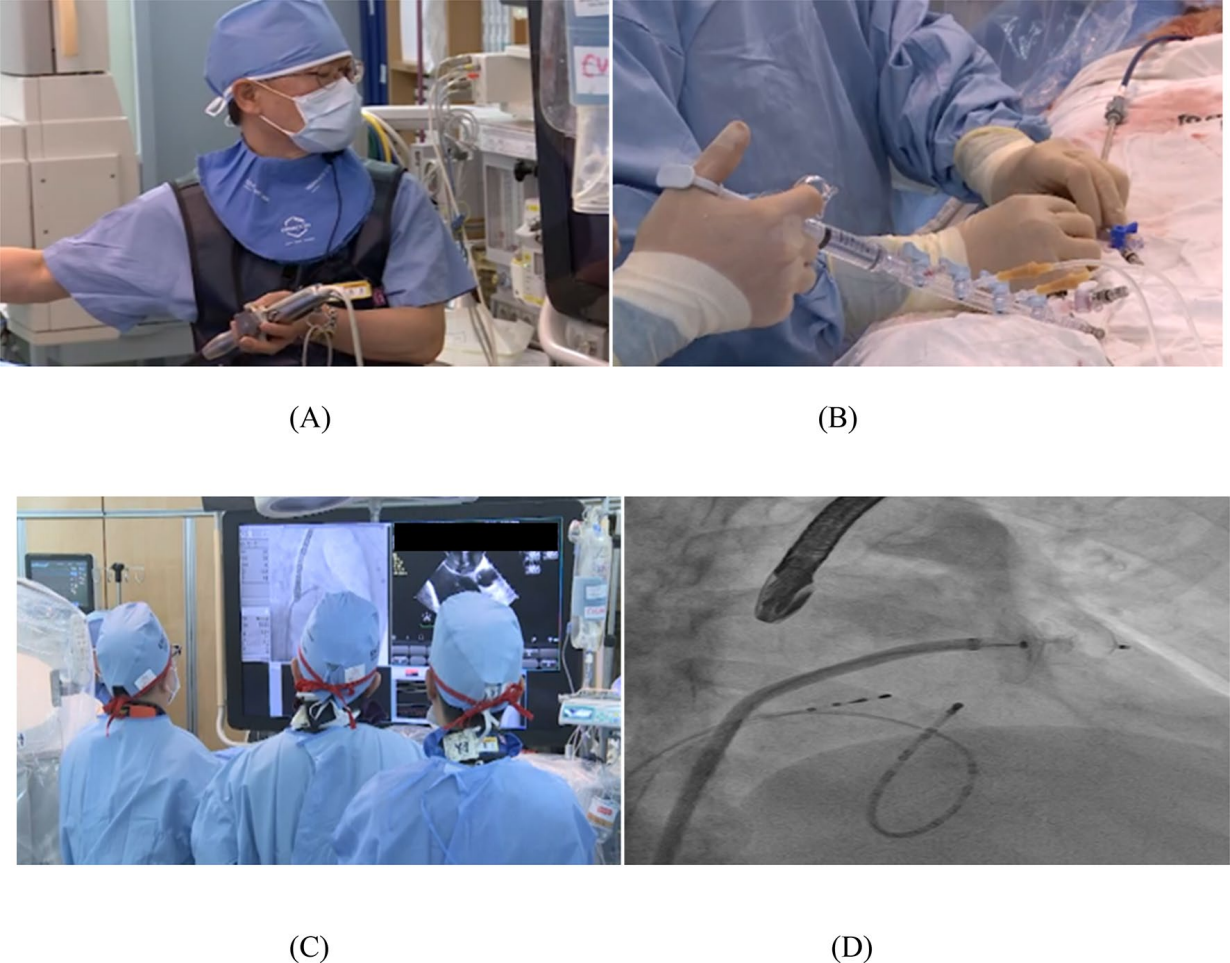
LAAO was performed using a device of the predicted size. (**A**) A cardiologist performing TEE during the procedure. (**B**) Another cardiologist injecting saline, medicine, and medium contrast through the manifold. (**C**) Checking of fluoroscopy and TEE in real time during the procedure. (**D**) Confirmation of the device insertion predicted by fluoroscopy correctly into the LAA. *LAA* left atrial appendage, *LAAO* left atrial appendage occlusion, *TEE* transesophageal echocardiography.

After producing the 3D-printed LAAO rehearsal phantom, a cardiologist simulated LAAO with the phantom. The clinician predicted the size of the LAAO device in the phantom simulation before the procedure and confirmed whether or not the size of the device used for the actual patient procedure was matched. In addition, the location and shape of the anatomical structures around the LAA were also confirmed (Fig. 7).

## Results

The LAAO 3D segmentation and 3D modeling process from CT images to an STL model as performed by an experienced operator consumed approximately 1–2 h. The export of the reconstructed mesh surface to the STL file took just a few minutes. However, most of the time was spent in 3D printing and post-process. It took 1–2 d to print as thinly as possible. Therefore, it took at least 3 d for the overall procedure, from receiving the patient's images to finishing the phantom.

A pilot study to determine 3D printing technology and materials was conducted to produce the rehearsal simulation phantoms. As a result of measuring 95A shore hardness by thickness using the Vernier calipers. Consequentially, although the material itself is flexible, FDM using TPU is not suitable for rehearsing LAAO because the thermoplastic material does not stretch well. It was also difficult to check the location of the device due to its opaqueness.

The final phantom was produced using an SLA 3D printer as an LAAO simulation phantom. The material property of the phantom needs elasticity to withstand device expansion and transparency to visually check the position of the device. Therefore, the LAAO rehearsal phantom was made using an SLA printer (X-Fab, DWS, Vicenza, Italy) with Flexa693, a photopolymer resin. Based on the material test results, the rehearsal phantom was printed with a thickness of 0.8 mm to maintain suitable physical properties through a 10-min UV curing process.

The corresponding landmarks between the original STL model and the printed phantoms were compared with the physical measurements and were evaluated using a Bland–Altman plot (Fig. 6). As a result, the determined limits of agreement were from − 1.8 to 1.6 mm (mean ± SD, 0.45 ± 0.37 mm). In addition, the greatest difference of the measured landmarks between the observers showed in the vertical zone. Among them, one patient showed a difference from the average value of up to 3.84 mm.

In addition, the rehearsal simulation for LAAO was evaluated by two expert cardiologists. The cardiologists predicted the LAAO device size for a total of 10 patients by using rehearsal simulation in consensus. In addition, the correctness of the predicted device sizes was evaluated by actual device sizes used in the actual procedure. The prediction accuracy for a total of 10 patients was 100%. The cardiologist performed LAAO using the predicted device. In addition, accurate 3D printing simulation was possible regardless of various LAA morphologies (Fig. 7 and Table 3). Informed consent was obtained for the publication of identifying images 7(A-C).

**Table 3:** Rehearsal simulation results of 10 patients.

	Morphology	Predicted device size (mm)	Inserted device size (mm)
PT 1	Cauliflower type	34	34
PT 2	Windsock type	28	28
PT 3	Chicken wing type	25	25
PT 4	Windsock type	28	28
PT 5	Cauliflower type	31	31
PT 6	Windsock type	25	25
PT 7	Windsock type	34	34
PT 8	Cauliflower type	34	34
PT 9	Windsock type	28	28
PT 10	Cauliflower type	25	25

*PT* patient.

## Discussion

LAAO is a structural intervention performed by checking certain regions, based on the medical image, during the procedure. TEE performed in the LAAO procedure makes image acquisition easy and fast, but it is difficult to accurately measure the 3D form and understand the shape. The lobe diameter measured by CT and TEE is measured assuming that the LAA is a cylinder. However, since the lobe has a thickness of 7.5–10 mm, there is a difference between the proximal landing and distal landing zone diameters when the lobe is inserted the LAA. In addition, because the cross-section of LAA is elliptical rather than circular, there can be long and short diameters in one plane. However, this depends on the plane being measured; thus, it could differ from the real one. To minimize these problems, the rehearsal with the 3D printing phantom before the procedure was evaluated while the device was inserted. In this way, the shape of the device after insertion (shape-stable, hockey puck-unstable/undersized, or strawberry-unstable/oversized) could be evaluated, and the stability of the device position after insertion could be assessed through a tug test to actually check the line of the device axis and the LAA neck axis. In addition, the position of the left circumflex artery (LCx) can be evaluated, and the relationship between the lobe and the LCx could be confirmed after the actual insertion. The actual structure can be judged in terms of whether the final device covers the appendageal ridge well or whether it does not invade the mitral valve.

Still, the application of 3D printing technology requires more cost and time, so it should be considered and applied reasonably. Flexible and transparent materials were selected in order to simulate the phantom with materials having properties similar to those of human tissue, considering the reasonable cost for manufacturing patient-specific phantoms. TPU of FDM 3D printer is a polymer-based material with some flexibility. Compared to SLA materials, this is not as flexible and transparent, so it could not be applied to actual rehearsals. Therefore, in this study, we used the flexible material of SLA has relatively good elasticity and transparency enough to see the positioning of the LAAO device from the outside, which could be a novelty of our method. In addition, the implementation of the physical properties of the actual human anatomical structure was attempted in order to show that the handling of the procedure can be similar. The material was decided upon by collecting the opinions of two cardiologists, referring to the shore A hardness of 3D printing materials with a texture similar to that handled in the actual treatment. Although it is difficult to apply this study to actual clinical practice right now due to costs or healthcare reimbursement, this method with 3D printing technology could have a potential to supplement current medical cares like an educational simulator for medical students and intervention fields with high image dependence.

This study had several limitations. First, only two medical doctors evaluated the suitability of the materials. In addition, despite an estimated 3-y study, the number of LAAOs was not sufficiently large, with only 10 patients enrolled. Therefore, there is a need to evaluate this phantom with more anatomical diversity of various kinds of LAAO patients in future studies. It should also be evaluated by the various cardiologists and by collecting various opinions through questionnaires. Second, it was difficult to accurately implement the texture of the actual human body with the currently available 3D printing materials. Therefore, to reflect physical properties more similar to that of the actual human body, it is necessary to develop silicon and similar 3D printing materials. Third, there was a limitation in the measurements performed for comparing the accuracy between the STL file and the 3D-printed phantom. There were outliers where a value outside the 95% confidence range appeared, as shown in Fig. 6. To make it similar to the physical properties of the actual heart tissue, it was manufactured to be as thin and flexible as possible; thus, the measurement value may not be constant, depending on the operator who uses the Vernier caliper when measuring. In particular, the landmark C in Fig. 5 is a measurement of the vertical area of the orifice, which was very difficult to measure using Vernier calipers due to the shape of the area and the material characteristics of the phantom. However, in the actual procedure, the accuracy of the device size prediction of the 3D printing phantom was very high because a circular device that filled in the LAA orifice was inserted. Fifth, the size of the LAA in the CT image may not have been fully reflected in some cases, depending on the patient's condition at the time of the CT scan. Therefore, CT scans prior to the procedure should be performed with caution. In conclusion, this rehearsal simulation using a 3D-printed LAAO phantom more accurate device size selection is possible because the 3D shape and architecture can be evaluated using the 3D-printed phantom, compared to the conventional method that predicted the device size using only CT and TEE.

## Data Availability

The datasets used and/or analyzed during the current study available from the corresponding author on reasonable request.
